# Scavengers on the Move: Behavioural Changes in Foraging Search Patterns during the Annual Cycle

**DOI:** 10.1371/journal.pone.0054352

**Published:** 2013-01-23

**Authors:** Pascual López-López, José Benavent-Corai, Clara García-Ripollés, Vicente Urios

**Affiliations:** 1 Vertebrates Zoology Research Group, CIBIO Research Institute, University of Alicante, Alicante, Spain; 2 Cavanilles Institute of Biodiversity and Evolutionary Biology, University of Valencia, Paterna, Valencia, Spain; University of Southern Denmark, Denmark

## Abstract

**Background:**

Optimal foraging theory predicts that animals will tend to maximize foraging success by optimizing search strategies. However, how organisms detect sparsely distributed food resources remains an open question. When targets are sparse and unpredictably distributed, a Lévy strategy should maximize foraging success. By contrast, when resources are abundant and regularly distributed, simple Brownian random movement should be sufficient. Although very different groups of organisms exhibit Lévy motion, the shift from a Lévy to a Brownian search strategy has been suggested to depend on internal and external factors such as sex, prey density, or environmental context. However, animal response at the individual level has received little attention.

**Methodology/Principal Findings:**

We used GPS satellite-telemetry data of Egyptian vultures *Neophron percnopterus* to examine movement patterns at the individual level during consecutive years, with particular interest in the variations in foraging search patterns during the different periods of the annual cycle (i.e. breeding vs. non-breeding). Our results show that vultures followed a Brownian search strategy in their wintering sojourn in Africa, whereas they exhibited a more complex foraging search pattern at breeding grounds in Europe, including Lévy motion. Interestingly, our results showed that individuals shifted between search strategies within the same period of the annual cycle in successive years.

**Conclusions/Significance:**

Results could be primarily explained by the different environmental conditions in which foraging activities occur. However, the high degree of behavioural flexibility exhibited during the breeding period in contrast to the non-breeding period is challenging, suggesting that not only environmental conditions explain individuals' behaviour but also individuals' cognitive abilities (e.g., memory effects) could play an important role. Our results support the growing awareness about the role of behavioural flexibility at the individual level, adding new empirical evidence about how animals in general, and particularly scavengers, solve the problem of efficiently finding food resources.

## Introduction

**Figure 1:**
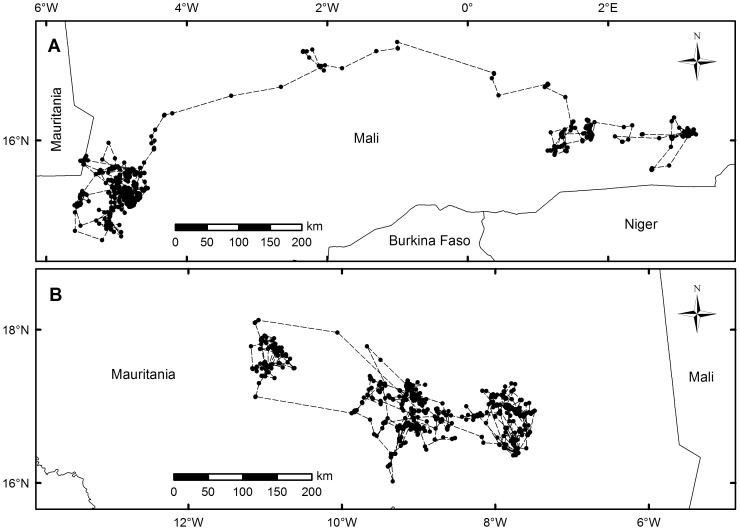
Comparison of Lévy and Brownian motion. Example of two tracks of two different Egyptian vultures (*Neophron percnopterus*) recorded by GPS satellite telemetry at two-hour intervals corresponding to a Lévy foraging search pattern (A) and Brownian random motion (B).

Optimal foraging theory predicts that animals will tend to maximize the success of finding food resources by optimizing search strategies [1]. How organisms detect sparsely distributed target food resources with little or no previous knowledge about where they are located is currently an object of scientific debate [2]–7. If prey are an abundant, predictable resource, a random “blind” search strategy (i.e. the so-called Brownian movement search pattern) would be sufficient to solve the problem of fulfilling energy requirements [8], [9]. However, prey are not usually randomly distributed in nature. Frequently, food resources are sparsely and unpredictably distributed and thus theory predicts that under these circumstances a search strategy that maximizes finding encounters of target resources should be selected [10]. The Lévy foraging hypothesis postulates that animals would follow so-called Lévy flights as an optimal search strategy [8], [11]. In brief, Lévy foraging patterns are a specialized random search strategy characterized by scale invariant fractal movement trajectories exhibiting a combination of an elevated number of small displacements (steps) connected by long distance relocation movements [3], [8] (Figure 1). Mathematically, in pure random Brownian motion the mean squared displacement from the starting point increases linearly with time (i.e. namely a normal diffusion process in physics), whereas it increases faster than linearly in a Lévy flight (i.e. leading to anomalous diffusion or super-diffusion) [10]. In both cases, the distribution of steps is drawn from a right skewed distribution [4]. The steps in a Lévy foraging pattern is drawn from a probability density function P(*l*)≈*l* ^−μ^, where *l* is the move-step length and *μ* the power-law coefficient. This probability distribution (the so-called Pareto-Lévy distribution) shows a power-law tail with 1<*μ*≤3, and theory predicts that the optimal search strategy appears when the power-law exponent approaches *μ*≈2 [9], [12]. By contrast, when *μ*>3, the shape of the tail changes and the motion approaches to a Brownian random walk.

There is empirical evidence that very different groups of organisms exhibit Lévy flight movement patterns, from bacteria [13], insects [14], [15], fishes [3], [12], [16], birds [4], [17], [18], and mammals [19], [20], including humans [21]–[23]. However, the shift from a Lévy to a Brownian search strategy has been suggested to depend on internal factors such as sex [19], and external factors such as prey density [13], [16] or environmental context [3], [20]. Although some researchers have questioned the extension of Lévy search pattern in nature [2], [24] and the conditions in which it emerges [5]–[7], there is a general agreement about the importance of seeking for optimal search patterns and its role as a natural force driving the evolution of free-living organisms [4].

The development of telemetry and the improvement in our understanding of optimal foraging theory have gone hand in hand since their beginnings [17]. However, recent advances in “biologging” technologies (i.e. the use of miniaturized animal-attached tags for studying animal's movements, behaviour, physiology and/or environment) have facilitated dramatic advances in the study of the spatial ecology of organisms [25], [26]. Nowadays, researchers can retrieve data across broad spatial and temporal scales, providing insight into how animals solve the problem of finding food resources. This is critical for the analysis of optimal foraging strategies (i.e. those that maximize biological fitness as well as minimizing energy expenditure). Oddly, up to now most studies assume that the response of animals to this problem occurs at the species level (i.e. all individuals by the mere fact of being of the same species would use similar foraging strategies under similar environmental conditions) [3], [4], with little attention to the response of animals at the individual level. Taking into account that natural selection operates mainly at the level of the individual [27], the study of individuals' responses becomes necessary, as the study of the spatial context in which the different foraging strategies occur [3], [16], [19], [28].

In this paper we used data of six Egyptian vultures (*Neophron percnopterus*) tracked by satellite telemetry to analyse movement patterns at the individual level over consecutive years. In particular we focused our analysis on the variation in foraging search patterns throughout the different phases of the annual cycle (i.e. breeding vs. non-breeding periods). The Egyptian vulture is long-lived medium-sized scavenger distributed along the Palearctic region, where it is threatened with extinction throughout its range, owing to a recent and extremely rapid population decline [29], [30]. Although it can be an opportunistic species, occasionally taking small mammals, birds and reptiles, this species feeds mainly on carrion [31], [32]. A peculiarity of the species is that it feeds on mammal faeces, primarily of ungulates, to obtain carotenoid pigments which are responsible for the colouration of the facial skin [32]. Egyptian Vultures roost communally on large trees and cliffs placed close to suitable foraging areas, which include dump sites, vulture restaurants and both intensive and extensive livestock farms. Continental populations are migratory, travelling from their European breeding grounds to the wintering areas located in the Sahel region of Africa [32], [33]. Consequently, the species' life-cycle takes place in habitats subject to very different levels of human alteration.

Scavenger species have evolved under a context of unpredictability with respect to trophic resources [34]. By nature, carrion is unpredictable both in space and time [35]. Therefore, the study of the spatial ecology of migratory scavengers can help us to gain deeper insight into optimal foraging strategies. Recent papers have shown how spatial heterogeneity in resources distribution (i.e. food) promotes changes in birds' behaviour, as a response to variations in environmental conditions between summering and wintering grounds [36] and between different habitats [3]. Taking into account human-induced habitat alterations and the rapid change in ecosystems management, mainly in developing countries [37], the study of the individual spatial response of migratory scavengers to changes in the distribution and availability of feeding resources during the annual cycle can help to improve our understanding about the optimal foraging strategy into a general dynamic context. To this end, using a GPS-based satellite-tracking dataset we specifically checked: i) the foraging strategies adopted by Egyptian vultures at the individual level; and ii) their variation during the annual cycle (i.e. if foraging strategies are different between breeding and non-breeding periods). We finally discuss the role of behavioural flexibility at the individual level and how human-induced changes in landscape composition and the concomitant changes in feeding regimes might disturb the movement patterns and the foraging strategies of scavengers in a broad context.

## Materials and Methods

### Study animals

Six adult Egyptian vultures were captured at the end of the breeding season from 2007 to 2009 at two vulture restaurants located in Castellón and Guadalajara provinces (Spain) and artificial feeding stations located within breeding territories. All measures are given in average ± standard deviation. Birds weighted 1932±208 g when were trapped. Birds were sexed by molecular methods [38]. Two birds were males (transmitter's code: #80420 and #89731) and four birds were females (#75657, #75659, #80419 and #89730). A 45 g solar-powered GPS tag from Microwave Telemetry Inc. was fixed to their backs using a harness sewn on with a cotton ribbon, designed to ensure that the harness would fall from the bird at the end of the tag's life. The mass of the equipment, including the harness, metal ring and tag, was less than 3% of the bird's body mass, which is within recommended limits [39]. The GPS tags were programmed to obtain GPS fixes every two hours on a 16 hours ON/8 hours OFF duty cycle for the breeding and non-breeding periods. Data were retrieved and managed using the Satellite Tracking and Analysis Tool [40]. Specific details about birds trapping and marking methods are available in [33].

### Ethics statement

Birds were trapped on public land and handled under veterinary supervision. Corresponding permissions were granted by the Spanish regional administrations (“Dirección General de Gestión del Medio Natural, Conselleria de Medi Ambient, Aigua, Urbanisme i Habitatge, Generalitat Valenciana” and “Consejería de Agricultura y Medio Ambiente, Junta de Comunidades de Castilla-La Mancha”, Spain).

### Movement data

We recorded GPS positions every two hours during both breeding and non-breeding periods from 2007 to 2012 (see electronic supplementary material, Table S1). GPS fixes were preliminary filtered, excluding erroneous locations (i.e. with 0 – 0 coordinates) and migratory periods. A step was defined as an interval in which any or both of the coordinates in two consecutive samples differed [41], and step length was calculated as the Euclidean distance between two consecutives positions. Distances below 25 m were eliminated because they were below the nominal accuracy of the GPS. Only diurnal distances recorded from 6:00 to 21:00 hours (Greenwich Mean Time) were considered. Nocturnal movements were excluded from analyses since Egyptian vultures do not forage during the nighttime [33]. Thus, for each dataset (i.e. individual-year-period) we obtained the distribution of move-step-lengths (the distance travelled between consecutive time intervals) that were used in subsequent analysis.

### Model fitting

We fitted the distribution of move-step-lengths to two random foraging strategies: Lévy and Brownian walks. A Lévy walk strategy was tested by fitting observed data to the probability density function of a truncated Pareto distribution (TP), whereas the Brownian walk strategy was tested using the probability density functions of a truncated exponential distribution (TEXP) (see electronic supplementary material, Table S2 and Appendix S1). Both distributions were truncated because natural movement data are inevitably bounded [4]. Recently, hyper-exponential distributions have been proposed to explain a likely Brownian walk strategy [7]. Therefore, in order to account for this new proposed candidate distribution, a Brownian walk was also tested by using a hyper-exponential distribution composed of two weighted exponential functions [7] (see electronic supplementary material, Table S2). This function was called a composite Brownian walk (CBW). Although other more complex hyper-exponential distributions have also been proposed for explaining Brownian motion [7], composed of a combination of multiple exponential functions, they were not considered in this study because they may lack biological meaning (see [6] for a complete discussion about this).

Parameters estimation of power-law distributions has substantially advanced in the last decade [42]. Although several approaches have been proposed, Maximum Likelihood Estimation (MLE) has been demonstrated to be the most accurate method for parameter estimation in right-skewed distributions (e.g. Pareto and Exponential families) [42], [43]. Here, we followed the MLE methodology as described in [44], which consists in increasing iteratively the values of x_min_ taken from each dataset (i.e. individual-year-period) and then estimating the parameters of the equation of the corresponding candidate model (TP, TEXP and CBW). In our case, x_max_ was defined as the maximum value observed in the move-step-lengths distribution dataset [7].

Then, for each data set (i.e. individual-year-period) we obtained as many sub-sets with different number of step lengths as different values of x_min_. However, unlike [44], we did not compute all possible values of x_min_ to allow inclusion of a substantial amount of data in the right tail of the move-step-lengths distribution [3], [4]. To this end, we stopped the fitting algorithm when it reached a fixed percentile of the data (in our case 75%, 80% and 85%), thereby allowing that at least 25%, 20% and 15% sub-set of the observed move-step-lengths distribution were included in the right tail, respectively. We did this in order to avoid fitting a very small sub-set of the data as a result of large values of x_min_ [3], [4]. Accordingly, we ensured that a minimum sample size of move-step-lengths was fitted to the tail of the candidate distribution (i.e. including long displacements that are crucial to discriminate between Lévy and Brownian motion).

MLE equations used for distribution fitting (see electronic supplementary material, Table S3) were solved numerically maximizing the log-likelihood function of the candidate model (i.e. TP, TEXP and CBW). To test for a Lévy foraging pattern, we solved numerically the exponent *μ* within the range 1<*μ*≤3 in the TP distribution. This was done because values of *μ*≤1 do not correspond to distribution that can be normalized [13], [45] and values of *μ*>3 do not correspond with a Lévy foraging pattern [45] (see electronic supplementary material, Appendix S2).

We tested the goodness of fit (GOF) of the models with the corrected δ-Kolomogorov-Smirnov (K-S) test [46]. This method tested the maximum distance between the observed and the estimated cumulative distribution function. Equations used for calculating cumulative distribution functions are summarized in electronic supplementary material (Table S2).

### Selection of the best parameters for each model

Firstly, using the K-S test we excluded combinations of parameters that did not fit significantly in statistical terms [45], [47]. Secondly, within each data set (i.e. individual-year-period) for each of the candidate models (TP, TEXP and CBW), we selected the best fit among the different sub-sets of data (i.e. defined by different values of x_min_). To this end, for each sub-set we calculated the corrected goodness of fit index (GOF_cor_) as presented in [4] to take into account the trade-off between goodness of fit and number of step-lengths. This index takes into account the K-S statistic (D) penalizing fittings with a reduced number of steps [4] as follows:
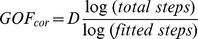
The lower this index the better the fit of the candidate model (TP, TEXP and CBW). GOF_cor_ allows rejecting groups of parameters that fit well to a small data sub-set in favour to group of parameters with a slightly worse goodness of fit, but fitted to a sub-set of data with higher number of step lengths [4]. This was done because increasing the number of steps increases the accuracy of the analyses [48]. At the end of this stage, we obtained the best fits for each tested model (TP, TEXP and CBW) (see electronic supplementary material, Appendix S2), plotting the best fitting models (TP, TEXP and CBW) in a log_10_ rank (y-axis) against log_10_ step-length (x-axis) using the full set of observations.

### Model selection

Three different scenarios were considered: i) when none the three models yielded a significant fit (i.e. neither Lévy nor Brownian motion describes the observed foraging search pattern; hereafter codified as “NONE”); ii) when only one model fitted significantly, thus it was the selected one (e.g. TP, EXP or CBW); and iii) when two or three models yielded significant results. Then, we applied model selection criteria to rank the competing models and thus select the model that best supported the data by means of a “truth-table” as described in [4] (see electronic supplementary material, Appendix S2). To do that we selected the two models that showed the best/lowest GOF_cor_ (e.g. model 1 and model 2) to evaluate the trade-off between goodness of fit and the number of steps used for fitting the model. Next, we calculated the Akaike weights (AIC_w_, [49], [50]) values for model 1 and its competing model (see electronic supplementary material, Table S3). The competing model was obtained by fitting the model 2 to the same sub-set of data (defined by the x_min_ value) with that used in the model 1. We repeated the reverse situation (i.e. model 2 vs. its competing model). Finally, we applied a “truth-table” as described in [4] in order to evaluate the trade-off between goodness of fit and complexity of the model (i.e. number of parameters) (see electronic supplementary material, Appendix S2). In summary, for each data set (i.e. individual-year-period) we finally tested the occurrence of a Lévy or a Brownian foraging pattern, or the absence of both strategies.

### Statistical analysis

Mann-Whitney and Kruskal-Wallis non-parametric tests were used to compare descriptive variables among individuals and between periods of the annual cycle. Differences among foraging patterns (Lévy, Brownian, or none) within each period of the annual cycle (breeding and non-breeding) were tested using a one-way contingency table with the Yates's chi-squared test [51]. Differences between breeding and non-breeding periods were tested using a two-way contingency table test [51]. In order to control the effect of individuals, we created a null distribution of the test statistic (χ^2^_sim_) through Monte Carlo simulations [51] permuting 10 000 times the winning models between periods within each individual [52]. P-value was calculated as the percentage of times that χ^2^_obs_ was strictly greater than χ^2^_sim_ [51]. Model fitting, model selection and statistical analyses were performed with the Matlab language of technical computing [53].

## Results

Six adult Egyptian vultures were instrumented for this study (see electronic supplementary material, Figure S1). Of these, one individual (bird #75657) was tracked over six years (from 2007 to 2012), four birds (#80419, #80420, #89730 and #89731) were tracked over four years (one from 2008 to 2011 and three from 2009 to 2012), and one bird (#75659) over three years (from 2009 to 2011)(see electronic supplementary material, Table S1). We obtained a total of 36276 GPS satellite fixes (average = 6046±1762 fixes/animal), for an average of 6.58±0.47 fixes/day, without differences among individuals (Kruskal-Wallis test, *H_5_*_,37_ = 1.817; *p* = 0.874). After filtering nocturnal data and distances recorded with a time lapse longer than two hours, we obtained 18990 valid steps for analyses (average = 3165±993 steps/animal) without differences among individuals (K-W test, *H_5,37_* = 2.833; *p* = 0.726). Considering the entire study period, we recorded on average 3.43±0.28 steps/day. 4398 steps were recorded during the breeding period (average = 244±132 steps/season) and 14592 steps were recorded during the non-breeding period (average = 768±64 steps/season). The number of GPS fixes and the number of steps differed significantly between seasons (Mann-Whitney U test, *Z_adj_* = 5.196; *p*<0.001; *Z_adj_* = 5.198; *p*<0.001). The duration of breeding and non-breeding periods was 140±58 days and 158±12 days, respectively, without significant differences between them (M-W test, *Z_adj_* = −0.897; *p* = 0.370). We did not test differences between sexes due to low sample size.

**Figure 2:**
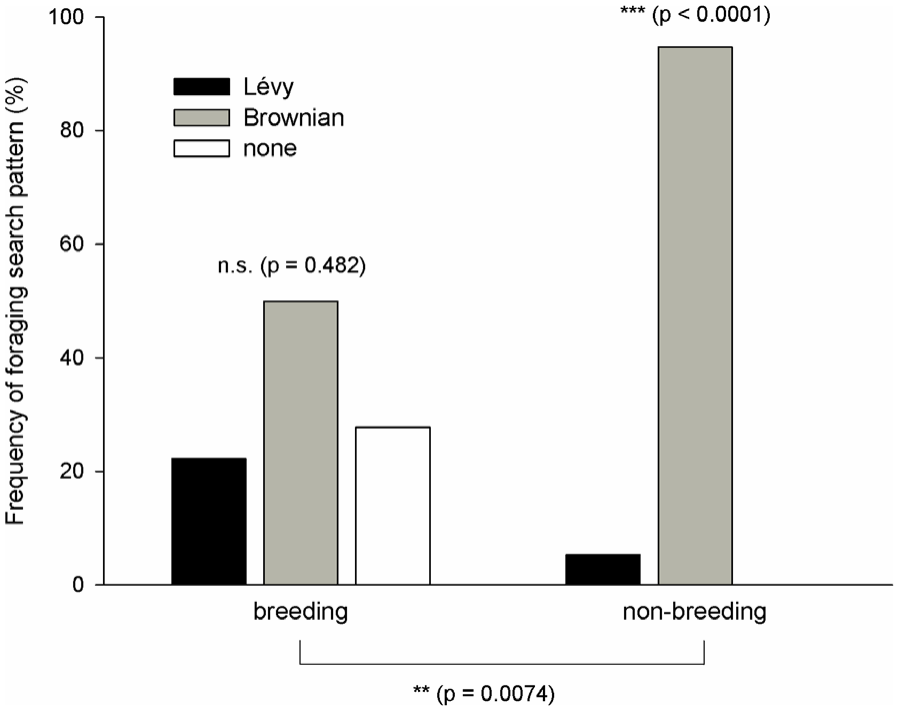
Occurrence of Lévy and Brownian behavior types of six Egyptian vultures tracked by GPS satellite telemetry during breeding (Europe) and non-breeding (Africa) periods. Differences within and between periods of the annual cycle were tested by means of a one-way contingency table with the Yates's chi-squared test and a two-way contingency table test, respectively. See text for more details.

**Figure 3:**
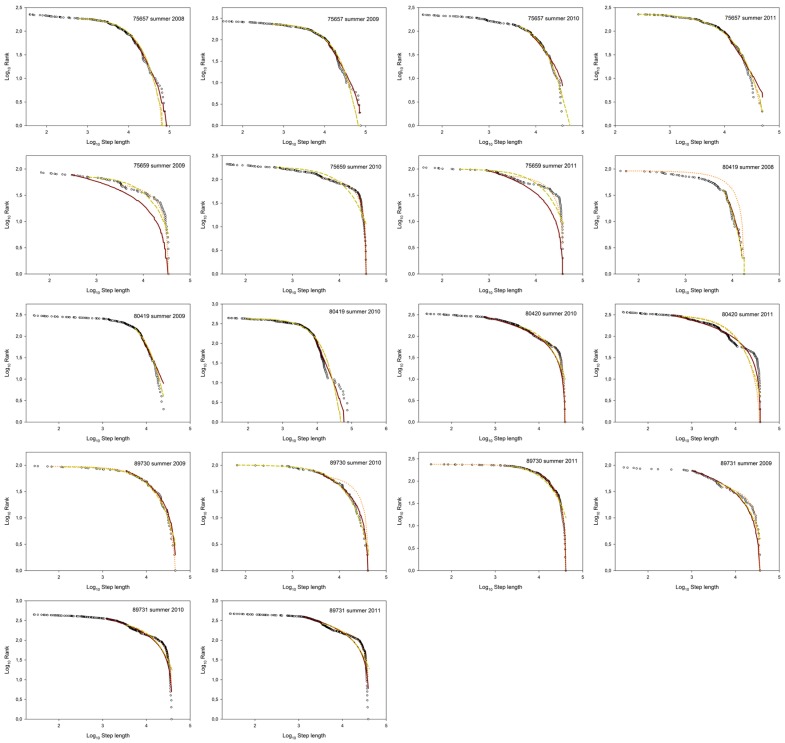
Ranked move-step-lengths plots with the best fitting models during the breeding period (Europe). Two random foraging strategies were considered: Lévy and Brownian motion. Lévy strategy was tested by fitting observed data (black circles) to the probability density function of a truncated Pareto distribution (red continuous line) whereas Brownian strategy was tested using the probability density functions of a truncated exponential distribution (orange dotted line) and a hyper-exponential distribution (green dashed line).

**Figure 4:**
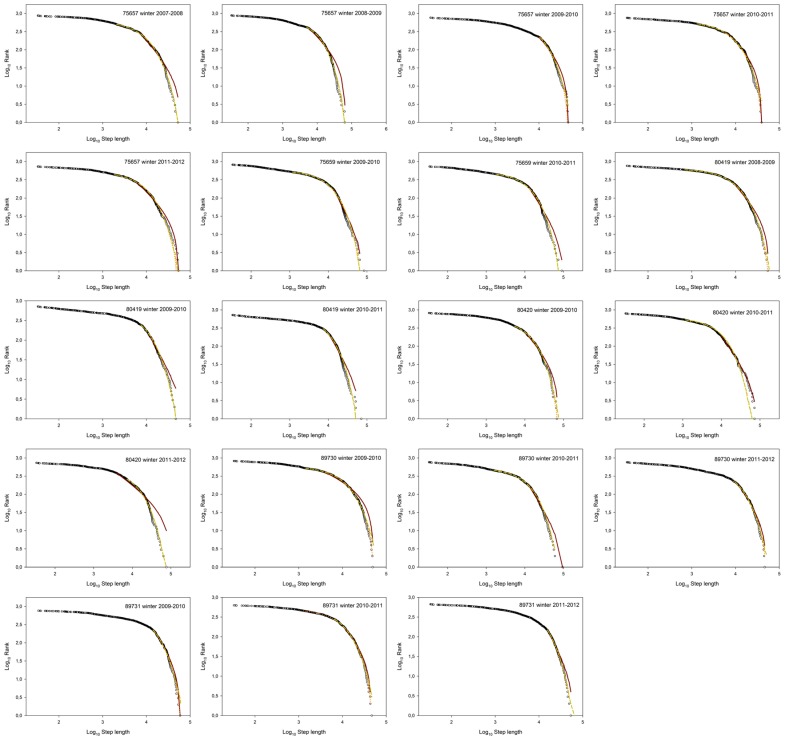
Ranked move-step-lengths plots with the best fitting models during the non-breeding period (Africa). Black circles show observed data. Three distributions are shown: truncated Pareto (red continuous line), truncated exponential (orange dotted line) and a hyper-exponential distribution (green dashed line).

During the breeding period nine trajectories fit a Brownian foraging search pattern (considering both TEXP and CBW), four trajectories fit a Lévy search pattern (TP), and five trajectories did not fit any pattern (Figures 2 and 3). By contrast, during the non-breeding period, almost all individuals followed a Brownian search strategy (18 trajectories) and only one followed a Lévy pattern (Figures 1, 2 and 4). Interestingly, these differences among foraging search strategies were consistently significant between periods (*p* = 0.0074) (Figure 2). In addition, significant differences in foraging patterns were found within the non-breeding period (*p*<0.0001), but not during the breeding period (*p* = 0.482) (Figure 2). Considering inter-annual variations, it is remarkable the individual behavioural flexibility was exhibited during the breeding period in Europe but was not during the wintering sojourn in Africa (see electronic supplementary material, Table S4). For example, there was one individual (bird #75657) that did not change its foraging search pattern (Brownian) throughout four consecutive breeding events. By contrast, the rest of individuals changed their foraging search strategies shifting from Lévy to Brownian motion in successive years during the breeding season. All results shown here were obtained using the 80% percentile of data in the fitting algorithm (see electronic supplementary material, Table S4). Remarkably, similar results were obtained either considering 75% percentile or 85% percentile in the analysis (see electronic supplementary material, Tables S5 and S6).

## Discussion

Scavenging has evolved in several different phylogenetic groups, from invertebrates to vertebrates [35]. However, a common factor of animals that have evolved to this strategy is their dependence on minimizing energy expenditure [54]. Carrion is unpredictable both in space and time and, as a consequence, scavengers need to seek an optimal searching strategy that maximizes successful encounter rates with their resources. Since soaring birds can fly over extensive areas with low energy cost, they are thus much more efficient at exploiting unpredictable food resources than other animals [54]. Up until now, most studies about foraging search strategies implicitly suggested that individuals of the same species should exhibit similar behaviour in response to similar conditions (but see [19] and [28]). However, there is a growing awareness that there is much more variation in individuals' responses (i.e. there is much more behavioural flexibility) than previously suggested (see reviews in [55]–[57]). The results shown here confirm previous theoretical suggestions on behavioural flexibility at the intraspecific level (e.g. [58]), adding new empirical evidence about how animals in general, and scavengers in particular, solve the problem of finding efficiently food resources.

Our results show that Egyptian vultures change their foraging search patterns during different periods of the annual cycle. Egyptian vultures follow a Brownian search strategy in their wintering sojourn in Africa, whereas they exhibit a more complex foraging search pattern at breeding grounds in Europe. This can be primarily explained by the different environmental conditions in which their activities occur. However, a detailed examination of the results shows that individuals shift from one search strategy to the other within the same period of the annual cycle in successive years (for example, individuals exhibiting a Lévy foraging strategy in summer 2009 and a Brownian strategy in summer 2010) (see electronic supplementary material, Table S4). This is challenging, given that it shows that not only environmental conditions explain individuals' behaviour but also suggest that individuals' cognitive abilities (e.g. memory effects) [59], [60] could play an important role that allows shifting between search strategies under similar environmental conditions. The decision-making behavioural mechanism by which animals detect the distribution of food resources (i.e. maximizing encounter probability) and thus select the optimal strategy still remains unknown, opening new avenues of future research that can also be applied to a broader ecological context in search theory such as encounters of predators and their prey, pollinator and plants or even mating encounters [11].

Migratory species need to cope with different environmental conditions over the entire annual cycle [61]. Interestingly, there are important differences in terms of landscape composition, climatic conditions and degree of human alteration between the European breeding grounds and the African wintering quarters [29], [37]. Food resources are sparsely distributed in the Sahel region, a transitional ecoregion between the Sahara desert and the southern tropical forest composed of semi-arid grasslands, savannahs, steppes, and thorn scrublands where traditional semi-nomad shepherds raise livestock in a system of transhumance [62]. This gives rise to environmental conditions where food resources can occur nearly randomly, a situation which is taken advantage of by Egyptian vultures [29], [36]. Consequently, it is not surprising that under these conditions, Egyptian vultures chose a Brownian search strategy, thus maximizing encounter rate. Interestingly, what is remarkable is the high level of site-fidelity to wintering areas year after year [33], which can probably be related to previous knowledge of the region plus the relative homogeneity of the landscape across vast extensions [37], [62], thus substantially enhancing the vultures' ability to move in these familiar areas.

By contrast, European breeding grounds have suffered different levels of human alteration. The most important change has occurred as a consequence of modification of livestock management regime [63]. Traditionally, scavengers have profited from extensive raising cattle distributed all over inner Spain. Furthermore, farmers have regularly dumped organic remains of dead cattle at specific feeding places called “vulture restaurants”, which were usually located in remote regions and close to traditional livestock areas, thus avoiding unnecessary expenditure in moving these remains to remote dumping sites. However, since the outbreak of the neurodegenerative disease in cattle, Bovine Spongiform Encephalopathy (BSE) and its variant in humans, Creutzfeldt-Jakob disease, mandatory regulations of the European Union led to the closure of uncontrolled existing vulture restaurants in order to avoid the likely risk of transmission (Regulation EC No. 1774/2002 of the European Parliament), allowing only dumping at a very few sites under sanitary supervision [63]–[65]. This change in cattle management regime has provoked a concomitant change in both the availability of food resources and its spatial distribution, thus increasing its predictability. Therefore, Egyptian vultures could have changed their foraging search pattern from a pure random strategy to an optimal search strategy when resources are neither abundant nor arbitrarily distributed. This outcome is reinforced by two facts: i) the pattern of aggregation of GPS satellite locations recorded at operating vulture restaurants; and ii) a crowding effect of Egyptian vultures at these predictable sources of food in accordance with field observations. In contrast to the situation in Africa, where Egyptian vultures are usually observed solitarily or in small groups integrated by few individuals, in Europe it is not exceptional observing up to several tens and even a hundred individuals concentrated at roosting places located close to suitable feeding areas ([33]; authors' pers. obs.). This is in agreement with the findings of [36] supporting that bird sociality at feeding grounds is closely linked to the pattern of spatial distribution and predictability of trophic resources.

Egyptian vultures are facultative scavengers that can profit from both unpredictable and predictable sources of food. However, it is an object of debate as to whether Egyptian vultures take profit of vulture restaurants as the primary food source at breeding areas or only forage there when other natural sources of food are not available. The Egyptian vultures tracked in this study showed high individual variation in the use of vulture restaurants during the breeding season. Whereas some individuals visited them regularly, others were recorded only at the beginning and at the end of the breeding season. The apparent lack of fit to neither a Brownian nor a Lévy strategy in 28% of move-step-lengths in summer shown in Figure 2 could be probably accounted for directed movements between breeding territories and predictable sources of food, which were exploited regularly by some individuals. A particular case of such behaviour was exhibited by bird #75657, which displaced every summer from its breeding place to a large vulture restaurant located 124 km away every two-three days (see electronic supplementary material, Figure S1). This behaviour was repeated every summer just before the onset of the breeding period and just prior starting the autumn migration to Africa, being only interrupted when the animal was incubating. Similar directed movements towards predictable sources of food were observed in the other birds (e.g. #80419, #80420, #75659 and #89731), of which the larger recorded movements were precisely those carried out between the breeding territories and some sparsely distributed vulture restaurant locations. Notwithstanding, although it is evident that Egyptian vultures profit from vulture restaurants, it should be noted that satellite-tracking data do not allow us to distinguish primary and secondary sources of food. If vultures would only use vulture restaurants to fulfill energy requirements, their tracks would be much more directional than those found here and thus no Brownian and Lévy search patterns would be detected. Therefore, the fit of individual trajectories to Lévy and Brownian search patterns shows that Egyptian vultures also exploit other food sources besides vulture restaurants during the breeding season. This has important implications from the conservation point of view, indicating that human actions have consequences in animals' behaviour.

Human activities can modify the availability and predictability of food resources. Therefore it would be expected that under a dynamically changing scenario animals are forced to adapt their foraging strategies in favour of those maximizing resource acquisition. This is interesting not only from a theoretical point of view, but also from the management perspective, especially of the predictable sources of food. Our results show that animals are able to change their foraging search patterns during the annual cycle under different environmental conditions. However, the degree of behavioural flexibility exhibited at the individual level demonstrates that not all animals shift between alternative foraging strategies in a similar way. Studying the threshold that provokes this shift in their behavioural response should be the object of further detailed analysis.

Comparing different environmental situations we hypothesize that the situation in Africa, where birds follow transhumant herds of cattle, could be considered primitive. By contrast, human-altered landscapes in Europe give rise to the modification in the predictability of resources, thus forcing birds to change to alternative foraging search patterns. It remains an open question if birds naturally grow under a predetermined program (e.g. Brownian search pattern) and whether they can modify it as their cognitive abilities or their knowledge of the environment increase. Future research should integrate the role of learning over the course of individual lifetime on the spatial response and movement ecology of birds. Understanding the role of individual behavioural flexibility in relation to other factors such as age [19] or social interactions [36] would improve existing theoretical models of movement. This would allow researchers to gain insight into the mechanisms underlying the behavioural ecology of organisms and would help managers to anticipate how changes in the distribution of food resources can affect their spatial ecology.

## Supporting Information

Figure S1 **Breeding and wintering locations of six Egyptian vultures tracked by GPS satellite telemetry.**
(JPG)

Table S1 **Summary information of the original GPS tracking dataset used to calculate flight profiles.**
(DOCX)

Table S2 **Equations of the probability density function (pdf) and the cumulative density function (cdf) for the truncated Pareto (TP), truncated exponential (TEXP) and hyper-exponential (CBW) functions.**
(DOCX)

Table S3 **Equations of the Log-Likelihood function (LLH) and Akaike Information Criterion (AIC) equations for the truncated Pareto (TP), truncated exponential (TEXP) and hyper-exponential (CBW) distributions.**
(DOCX)

Table S4 **Best fit parameters and model comparison analysis of move-step-length distribution recorded during the breeding and non-breeding periods using the percentile 80% of the move-step-length distributions.**
(DOCX)

Table S5 **Bests fit results and model comparison analysis using the percentile 75% of the move-step-length distributions.**
(DOCX)

Table S6 **Bests fit results and model comparison analysis using the percentile 85% of the move-step-length distributions.**
(DOCX)

Appendix S1 **Probability density function (pdf), cumulative density function (cdf) and complementary density function (CDF) for the Truncated Exponential distribution.**
(PDF)

Appendix S2 **Process diagram of the methods used in this study.**
(PDF)
