# Supplementary figures and images for: Scavengers on the Move: Behavioural Changes in Foraging Search Patterns during the Annual Cycle
Source: PLoS One. 2013 Jan 23;8(1):e54352. doi: 10.1371/journal.pone.0054352 (PMC3553087; doi:10.1371/journal.pone.0054352)

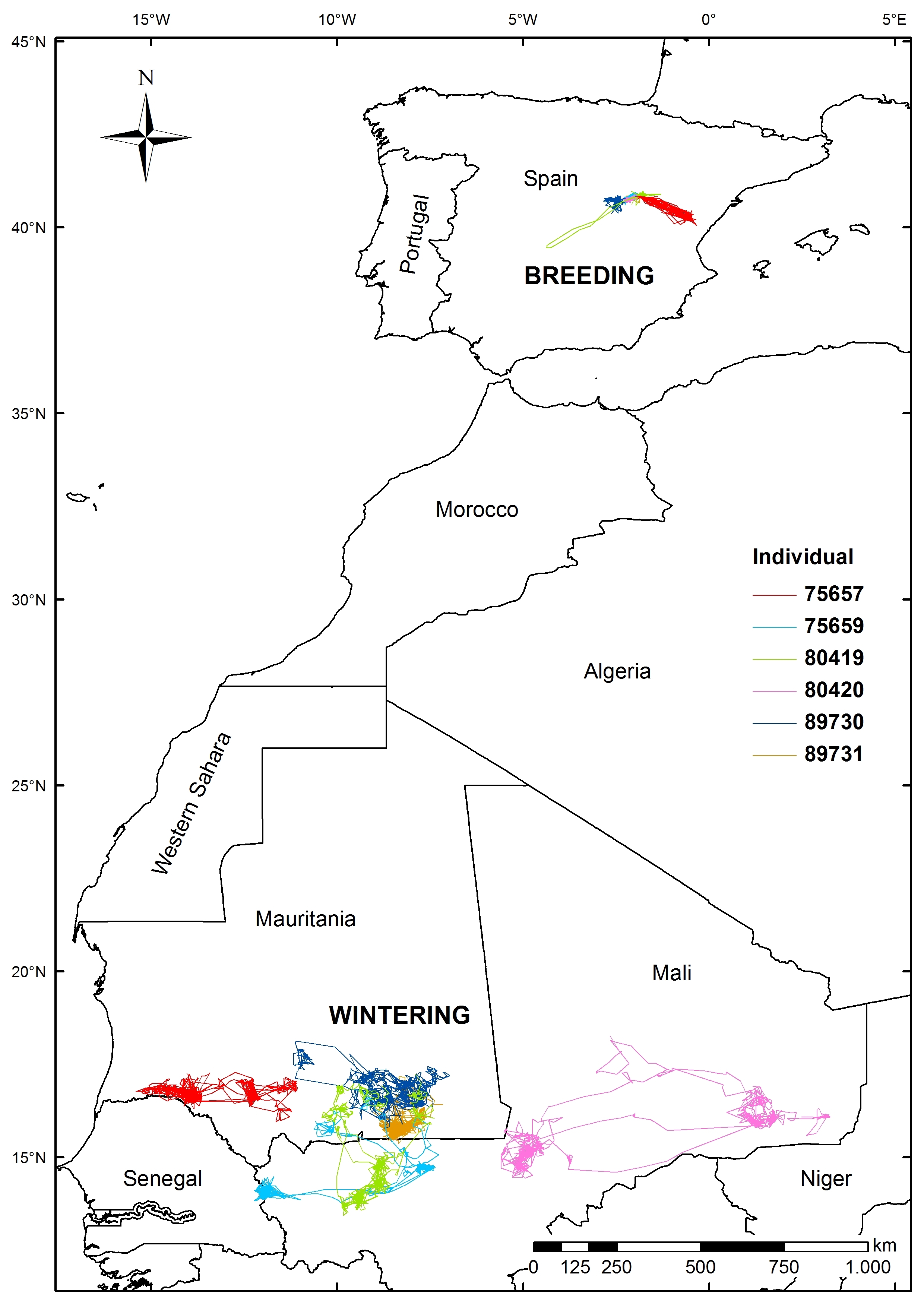

Supplement: Figure S1 — Breeding and wintering locations of six Egyptian vultures tracked by GPS satellite telemetry. (JPG) [file pone.0054352.s001.jpg]
