# Supplementary material for: Scavengers on the Move: Behavioural Changes in Foraging Search Patterns during the Annual Cycle
Source: PLoS One. 2013 Jan 23;8(1):e54352. doi: 10.1371/journal.pone.0054352 (PMC3553087; doi:10.1371/journal.pone.0054352)
Supplement: Table S1 — Summary information of the original GPS tracking dataset used to calculate flight profiles. (DOCX) [file pone.0054352.s002.docx]

**Table S1.** Summary information of the original GPS tracking dataset used to calculate flight profiles. Lags = GPS positions. MCP = surface calculated according to the minimum convex polygon.

| Individual | Period | Reference | Start | End | Days | Hours | Nº lags observed | Min step (Km) | Max step (Km) | Nº steps | MCP (km^2^) |
| --- | --- | --- | --- | --- | --- | --- | --- | --- | --- | --- | --- |
| 75657 | winter | 75657_wint_07_08 | 25/09/2007 | 29/02/2008 | 157 | 3784 | 1538 | 0.033 | 63.604 | 877 | 19811 |
|  | winter | 75657_wint_08_09 | 13/09/2008 | 22/02/2009 | 162 | 3898 | 1581 | 0.033 | 67.450 | 890 | 32936 |
|  | winter | 75657_wint_09_10 | 28/09/2009 | 04/03/2010 | 157 | 3762 | 1446 | 0.033 | 47.058 | 769 | 21750 |
|  | winter | 75657_wint_10_11 | 23/09/2010 | 27/02/2011 | 157 | 3764 | 1415 | 0.033 | 40.123 | 761 | 21737 |
|  | winter | 75657_wint_11_12 | 16/09/2011 | 26/02/2012 | 163 | 3912 | 1192 | 0.033 | 55.204 | 733 | 16601 |
|  | summer | 75657_sum_08 | 14/03/2008 | 02/09/2008 | 172 | 4118 | 454 | 0.027 | 90.751 | 232 | 3636 |
|  | summer | 75657_sum_09 | 17/03/2009 | 15/09/2009 | 182 | 4362 | 572 | 0.028 | 95.663 | 277 | 4663 |
|  | summer | 75657_sum_10 | 24/03/2010 | 10/09/2010 | 170 | 4080 | 496 | 0.027 | 53.590 | 230 | 3954 |
|  | summer | 75657_sum_11 | 23/03/2011 | 05/09/2011 | 166 | 3990 | 537 | 0.266 | 93.210 | 229 | 4309 |
| 75659 | winter | 75659_wint_09_10 | 20/09/2009 | 28/02/2010 | 161 | 3860 | 1572 | 0.033 | 86.808 | 823 | 92953 |
|  | winter | 75659_wint_10_11 | 24/09/2010 | 25/02/2011 | 154 | 3700 | 1435 | 0.034 | 102.967 | 732 | 67474 |
|  | summer | 75659_sum_09 | 30/07/2009 | 06/09/2009 | 38 | 909 | 152 | 0.027 | 34.693 | 91 | 444 |
|  | summer | 75659_sum_10 | 19/03/2010 | 08/09/2010 | 173 | 4144 | 417 | 0.028 | 37.622 | 219 | 745 |
|  | summer | 75659_sum_11 | 20/03/2011 | 23/07/2011 | 125 | 2994 | 210 | 0.028 | 39.200 | 109 | 349 |
| 80419 | winter | 80419_wint_08_09 | 02/10/2008 | 18/02/2009 | 139 | 3334 | 1344 | 0.033 | 59.684 | 769 | 45970 |
|  | winter | 80419_wint_09_10 | 18/09/2009 | 12/02/2010 | 147 | 3530 | 1331 | 0.033 | 86.759 | 719 | 27344 |
|  | winter | 80419_wint_10_11 | 28/09/2010 | 18/02/2011 | 143 | 3434 | 1337 | 0.033 | 72.940 | 725 | 37520 |
|  | summer | 80419_sum_08 | 14/08/2008 | 19/09/2008 | 36 | 862 | 161 | 0.027 | 17.882 | 94 | 113 |
|  | summer | 80419_sum_09 | 09/03/2009 | 04/09/2009 | 179 | 4288 | 626 | 0.027 | 55.577 | 309 | 667 |
|  | summer | 80419_sum_10 | 01/03/2010 | 10/09/2010 | 193 | 4630 | 915 | 0.027 | 87.261 | 452 | 8625 |
| 80420 | winter | 80420_wint_09_10 | 13/09/2009 | 26/02/2010 | 166 | 3982 | 1510 | 0.033 | 75.936 | 823 | 166678 |
|  | winter | 80420_wint_10_11 | 24/09/2010 | 25/02/2011 | 154 | 3697 | 1449 | 0.033 | 87.780 | 798 | 148076 |
|  | winter | 80420_wint_11_12 | 15/09/2011 | 18/02/2012 | 156 | 3741 | 1408 | 0.033 | 103.454 | 737 | 80284 |
|  | summer | 80420_sum_10 | 17/03/2010 | 08/09/2010 | 175 | 4204 | 647 | 0.027 | 39.832 | 335 | 985 |
|  | summer | 80420_sum_11 | 18/03/2011 | 04/09/2011 | 170 | 4072 | 690 | 0.027 | 37.440 | 368 | 873 |
| 89730 | winter | 89730_wint_09_10 | 31/08/2009 | 08/03/2010 | 189 | 4530 | 1846 | 0.033 | 51.693 | 831 | 22790 |
|  | winter | 89730_wint_10_11 | 30/08/2010 | 21/02/2011 | 175 | 4196 | 1661 | 0.033 | 104.240 | 768 | 48127 |
|  | winter | 89730_wint_11_12 | 09/09/2011 | 28/02/2012 | 172 | 4121 | 1584 | 0.033 | 52.389 | 768 | 22806 |
|  | summer | 89730_sum_09 | 09/07/2009 | 20/08/2009 | 42 | 1009 | 164 | 0.028 | 48.481 | 98 | 1642 |
|  | summer | 89730_sum_10 | 27/03/2010 | 13/08/2010 | 139 | 3344 | 213 | 0.066 | 41.938 | 101 | 1351 |
|  | summer | 89730_sum_11 | 22/03/2011 | 27/08/2011 | 158 | 3796 | 380 | 0.034 | 41.306 | 239 | 1641 |
| 89731 | winter | 89731_wint_09_10 | 19/09/2009 | 27/02/2010 | 161 | 3869 | 1536 | 0.034 | 60.939 | 768 | 17304 |
|  | winter | 89731_wint_10_11 | 27/09/2010 | 21/02/2011 | 147 | 3526 | 1268 | 0.033 | 46.942 | 629 | 8827 |
|  | winter | 89731_wint_11_12 | 25/09/2011 | 22/02/2012 | 150 | 3596 | 1348 | 0.033 | 64.377 | 672 | 15053 |
|  | summer | 89731_sum_09 | 29/07/2009 | 07/09/2009 | 40 | 964 | 145 | 0.028 | 37.289 | 92 | 249 |
|  | summer | 89731_sum_10 | 14/03/2010 | 05/09/2010 | 175 | 4194 | 848 | 0.027 | 39.310 | 448 | 430 |
|  | summer | 89731_sum_11 | 14/03/2011 | 12/09/2011 | 182 | 4372 | 848 | 0.027 | 40.953 | 475 | 841 |
