# Supplementary material for: Scavengers on the Move: Behavioural Changes in Foraging Search Patterns during the Annual Cycle
Source: PLoS One. 2013 Jan 23;8(1):e54352. doi: 10.1371/journal.pone.0054352 (PMC3553087; doi:10.1371/journal.pone.0054352)
Supplement: Table S2 — Equations of the probability density function (pdf) and the cumulative density function (cdf) for the truncated Pareto (TP), truncated exponential (TEXP) and hyper-exponential (CBW) functions. (DOCX) [file pone.0054352.s003.docx]

**Table S2.** Equations of the probability density function (pdf) and the cumulative density function (cdf) for the truncated Pareto (TP), truncated exponential (TEXP) and hyperexponential (CBW) functions. First column includes the range of data and parameter values over which it applies.

| Function | pdf | cdf | Source |
| --- | --- | --- | --- |
| TP  $0<a$ , $0<b$  $1<\mu\leq3$ | $\left( 1-\mu\right)\cdot{{(b^{1-\mu}-a}^{1-\mu})}^{-1}\cdot x^{-\mu}$ | $\frac{x^{(1-\mu)}- a^{(1-\mu)}}{b^{(1-\mu)}- a^{(1-\mu)}}$ | 1 |
| TEXP  $0<a$ , $0<b$  $0<\text{}$ | ${\frac{\text{}}{{e^{\text{-}\text{}\text{.}a}-e}^{\text{-}\text{}\text{.}b}} .}^{e^{\text{-}\text{}\text{.}x}}$ | $\frac{e^{\text{-}\text{}\text{.}a}}{{e^{\text{-}\text{}\text{.}a}-e}^{\text{-}\text{}\text{.}b}} -\frac{e^{\text{-}\text{}\text{.}x}}{{e^{\text{-}\text{}\text{.}a}-e}^{\text{-}\text{}\text{.}b}}$ | present study |
| CBW  $0<a$  $0<\text{}$  $0<\text{p}\text{ <1}$ | $p\text{. }\text{}_{1}\text{.}e^{-\text{}_{1}.\left( x-a \right)}+\left( 1-p \right).\text{}_{2}\text{.}e^{-\text{}_{2}.\left( x-a \right)}$ | $1-\left[ p\text{. }e^{-\text{}_{1}.\left( x-a \right)}+\left( 1-p \right).e^{-\text{}_{2}.\left( x-a \right)} \right]$ | 2 |

References:

1 White EP, Enquist BJ, Green JL (2008) On estimating the exponent of power-law frequency distributions. Ecology 89: 905-912.

2 Jasen VAA, Mashanova A, Petrovskii S (2012) Comment on “Lévy walks evolve through interaction between movement and environmental complexity”. Science 335: 918-c.
