# Supplementary material for: Scavengers on the Move: Behavioural Changes in Foraging Search Patterns during the Annual Cycle
Source: PLoS One. 2013 Jan 23;8(1):e54352. doi: 10.1371/journal.pone.0054352 (PMC3553087; doi:10.1371/journal.pone.0054352)
Supplement: Table S3 — Equations of the Log-Likelihood function (LLH) and Akaike Information Criterion (AIC) equations for the truncated Pareto (TP), truncated exponential (TEXP) and hyper-exponential (CBW) distributions. (DOCX) [file pone.0054352.s004.docx]

**Table S3.** Equations of the Log-Likelihood function (LLH) and Akaike Information Criterion (AIC) equations for the truncated Pareto (TP), truncated exponential (TEXP) and hyperexponential (CBW) distributions. First column includes the range of data and parameter values over which it applies. K is the number of parameters estimated for each model.

| Function | LLH | AIC | Source |
| --- | --- | --- | --- |
| TP  $0<a$ , $0<b$  $1<\mu\leq3$ | $n\log\left( \mu-1 \right)-n\log(a^{\left( 1-\mu\right)}- b^{\left( 1-\mu\right)})-\mu\sum_{i=1}^{n} \log x_{i}$ | $-2.{LLH}_{TP}+2 .K$  $K=3 \left\{ a, b, \mu\right\}$ | 1 |
| TEXP  $0<a$ , $0<b$  $0<\text{}$ | $\sum_{i=1}^{n} \log\left[ {\frac{\text{}}{{e^{\text{-}\text{}\text{.}a}-e}^{\text{-}\text{}\text{.}b}} .}^{e^{\text{-}\text{}\text{.}x_{i}}} \right]$ | $-2.{LLH}_{TEXP}+2 .K$  $K=3 \left\{ a, b, \text{} \right\}$ | present study |
| CBW  $0<a$  $0<\text{}$  $0\leq p \leq1$ | $\sum_{i=1}^{n} \log\left[ p\text{. }\text{}_{1}\text{.}e^{-\text{}_{1}.\left( x_{i}-a \right)}+\left( 1-p \right).\text{}_{2}\text{.}e^{-\text{}_{2}.\left( x_{i}-a \right)} \right]$ | $-2.{LLH}_{CBW}+2 .K$  $K=4 \left\{ a, p, \text{}_{1}, \text{}_{2} \right\}$ | 1 |

References:

1 Jasen VAA, Mashanova A, Petrovskii S (2012) Comment on “Lévy walks evolve through interaction between movement and environmental complexity”. Science 335: 918-c.
