# Supplementary material for: Scavengers on the Move: Behavioural Changes in Foraging Search Patterns during the Annual Cycle
Source: PLoS One. 2013 Jan 23;8(1):e54352. doi: 10.1371/journal.pone.0054352 (PMC3553087; doi:10.1371/journal.pone.0054352)
Supplement: Table S5 — Bests fit results and model comparison analysis using the percentile 75% of the move-step-length distributions. (DOCX) [file pone.0054352.s006.docx]

**Table S5A.** Bests fit parameters and model comparison analysis of move-step-length distribution recorded during the breeding period. Two foraging search patterns were considered: Lévy motion (truncated Pareto, TP) and Brownian motion (truncated exponential (TEXP) and hyperexponential (CBW)). Only significant fits are shown. Results were obtained using the percentile 75% of the move-step-length distributions. Abbreviations: N = number of steps, AIC_w_ = AIC weight, GOF_cor_ = corrected goodness of fit. Note that in each row there are only two AIC_w_ values corresponding to the two best competing models evaluated with the truth table. See Model selection in Methods for further details.

|  |  | Truncated Pareto (TP) | | | | | | Truncated Exponential (TEXP) | | | | | | Hyperexponential (CBW) | | | | | | | |
| --- | --- | --- | --- | --- | --- | --- | --- | --- | --- | --- | --- | --- | --- | --- | --- | --- | --- | --- | --- | --- | --- |
| Individual | Winner Model | N | Xmin | Xmax | µ | GOF_cor_ | AICw | N | Xmin | Xmax | λ | GOF_cor_ | AICw | N | Xmin | Xmax | P | λ_1_ | λ_2_ | GOF_cor_ | AICw |
| 75657_sum_08 | TEXP | 80 | 11.872 | 90.751 | 2.310 | 0.082 | -- | 186 | 0.602 | 90.751 | 0.074 | 0.059 | 0.75 | 186 | 0.602 | 90.751 | 0.497 | 0.079 | 0.071 | 0.056 | 0.25 |
| 75657_sum_09 | TEXP | 107 | 10.362 | 95.663 | 2.466 | 0.096 | -- | 233 | 0.536 | 95.663 | 0.084 | 0.054 | 0.73 | 233 | 0.536 | 95.663 | 0.599 | 0.088 | 0.079 | 0.054 | 0.27 |
| 75657_sum_10 | TEXP | 104 | 6.635 | 53.590 | 1.893 | 0.085 | -- | 129 | 4.305 | 53.590 | 0.104 | 0.036 | 0.85 | 128 | 4.394 | 53.590 | 1.000 | 0.107 | 0.096 | 0.042 | 0.15 |
| 75657_sum_11 | TEXP | 98 | 9.960 | 93.210 | 2.676 | 0.091 | -- | 226 | 0.381 | 93.210 | 0.096 | 0.043 | 0.73 | 229 | 0.266 | 93.210 | 0.702 | 0.100 | 0.089 | 0.043 | 0.27 |
| 75659_sum_09 | TEXP | -- | -- | -- | -- | -- | -- | 49 | 3.448 | 34.693 | 1.1*10^-6^ | 0.131 | -- | -- | -- | -- | -- | -- | -- | -- | -- |
| 75659_sum_10 | NONE | -- | -- | -- | -- | -- | -- | -- | -- | -- | -- | -- | -- | -- | -- | -- | -- | -- | -- | -- | -- |
| 75659_sum_11 | NONE | -- | -- | -- | -- | -- | -- | -- | -- | -- | -- | -- | -- | -- | -- | -- | -- | -- | -- | -- | -- |
| 80419_sum_08 | TP | 40 | 6.432 | 17.882 | 2.435 | 0.136 | 0.98 | -- | -- | -- | -- | -- | -- | 40 | 6.432 | 17.882 | 0.010 | 0.322 | 0.287 | 0.135 | 0.02 |
| 80419_sum_09 | TEXP | 123 | 6.887 | 55.577 | 3.000 | 0.069 | -- | 155 | 5.443 | 55.577 | 0.192 | 0.059 | 0.71 | 155 | 5.443 | 55.577 | 0.573 | 0.202 | 0.181 | 0.061 | 0.29 |
| 80419_sum_10 | NONE | -- | -- | -- | -- | -- | -- | -- | -- | -- | -- | -- | -- | -- | -- | -- | -- | -- | -- | -- | -- |
| 80420_sum_10 | TEXP | -- | -- | -- | -- | -- | -- | 99 | 8.408 | 39.832 | 1.2*10^-6^ | 0.106 | -- | -- | -- | -- | -- | -- | -- | -- | -- |
| 80420_sum_11 | NONE | -- | -- | -- | -- | -- | -- | -- | -- | -- | -- | -- | -- | -- | -- | -- | -- | -- | -- | -- | -- |
| 89730_sum_09 | TP | 78 | 3.493 | 48.481 | 1.047 | 0.056 | 1.00 | 95 | 0.068 | 48.481 | 0.062 | 0.059 | 1.00 | 93 | 0.124 | 48.481 | 0.502 | 0.077 | 0.069 | 0.062 | -- |
| 89730_sum_10 | CBW | 80 | 2.764 | 41.938 | 1.009 | 0.066 | 0.99 | -- | -- | -- | -- | -- | -- | 101 | 0.066 | 41.938 | 0.638 | 0.092 | 0.083 | 0.063 | 1.00 |
| 89730_sum_11 | TEXP | 145 | 10.461 | 41.306 | 1.001 | 0.060 | 1.00 | 239 | 0.034 | 41.306 | 0.038 | 0.033 | 1.00 | -- | -- | -- | -- | -- | -- | -- | -- |
| 89731_sum_09 | TP | 79 | 1.036 | 37.289 | 1.001 | 0.094 | 1.00 | 39 | 4.814 | 37.289 | 1.2*10^-6^ | 0.102 | 0.98 | -- | -- | -- | -- | -- | -- | -- | -- |
| 89731_sum_10 | NONE | -- | -- | -- | -- | -- | -- | -- | -- | -- | -- | -- | -- | -- | -- | -- | -- | -- | -- | -- | -- |
| 89731_sum_11 | NONE | -- | -- | -- | -- | -- | -- | -- | -- | -- | -- | -- | -- | -- | -- | -- | -- | -- | -- | -- | -- |

**Table S5B.** Bests fit parameters and model comparison analysis of move-step-length distribution recorded during the non-breeding period. Two foraging search patterns were considered: Lévy motion (truncated Pareto, TP) and Brownian motion (truncated exponential (TEXP) and hyperexponential (CBW)). Only significant fits are shown. Results were obtained using the percentile 75% of the move-step-length distributions. Abbreviations: N = number of steps, AIC_w_ = AIC weight, GOF_cor_ = corrected goodness of fit. Note that in each row there are only two AIC_w_ values corresponding to the two best competing models evaluated with the truth table. See Model selection in Methods for further details.

|  |  | Truncated Pareto (TP) | | | | | | Truncated Exponential (TEXP) | | | | | | Hyperexponential (CBW) | | | | | | | |
| --- | --- | --- | --- | --- | --- | --- | --- | --- | --- | --- | --- | --- | --- | --- | --- | --- | --- | --- | --- | --- | --- |
| Individual | Model | N | Xmin | Xmax | µ | GOF_cor_ | AICw | N | Xmin | Xmax | λ | GOF_cor_ | AICw | N | Xmin | Xmax | P | λ_1_ | λ_2_ | GOF_cor_ | AICw |
| 75657_wint_07_08 | TEXP | 294 | 7.141 | 63.604 | 2.317 | 0.058 | -- | 496 | 2.107 | 63.604 | 0.115 | 0.039 | 0.80 | 502 | 2.092 | 63.604 | 0.860 | 0.119 | 0.107 | 0.037 | 0.20 |
| 75657_wint_08_09 | TEXP | -- | -- | -- | -- | -- | -- | 418 | 4.981 | 67.450 | 0.098 | 0.027 | 0.87 | 419 | 4.930 | 67.450 | 1.000 | 0.099 | 0.089 | 0.030 | 0.13 |
| 75657_wint_09_10 | TEXP | -- | -- | -- | -- | -- | -- | 222 | 10.430 | 47.058 | 0.111 | 0.051 | 0.98 | 216 | 10.711 | 47.058 | 1.000 | 0.121 | 0.108 | 0.072 | 0.02 |
| 75657_wint_10_11 | TEXP | -- | -- | -- | -- | -- | -- | 489 | 1.592 | 40.123 | 0.120 | 0.039 | -- | -- | -- | -- | -- | -- | -- | -- | -- |
| 75657_wint_11_12 | TEXP | -- | -- | -- | -- | -- | -- | 434 | 1.882 | 55.204 | 0.116 | 0.037 | 0.86 | 434 | 1.882 | 55.204 | 0.508 | 0.124 | 0.111 | 0.032 | 0.14 |
| 75659_wint_09_10 | TEXP | -- | -- | -- | -- | -- | -- | 513 | 1.172 | 86.808 | 0.093 | 0.033 | 0.76 | 512 | 1.177 | 86.808 | 0.671 | 0.096 | 0.086 | 0.035 | 0.24 |
| 75659_wint_10_11 | TEXP | -- | -- | -- | -- | -- | -- | 451 | 1.764 | 102.967 | 0.080 | 0.036 | 0.76 | 451 | 1.764 | 102.967 | 0.569 | 0.084 | 0.076 | 0.036 | 0.24 |
| 80419_wint_08_09 | TEXP | -- | -- | -- | -- | -- | -- | 590 | 0.724 | 59.684 | 0.103 | 0.025 | 0.91 | 595 | 0.689 | 59.684 | 0.765 | 0.107 | 0.096 | 0.028 | 0.09 |
| 80419_wint_09_10 | TEXP | -- | -- | -- | -- | -- | -- | 245 | 7.523 | 86.759 | 0.137 | 0.048 | 0.71 | 246 | 7.484 | 86.759 | 0.558 | 0.144 | 0.129 | 0.047 | 0.29 |
| 80419_wint_10_11 | TEXP | -- | -- | -- | -- | -- | -- | 304 | 7.350 | 72.940 | 0.122 | 0.047 | 0.74 | 303 | 7.389 | 72.940 | 0.621 | 0.128 | 0.115 | 0.046 | 0.26 |
| 80420_wint_09_10 | TEXP | -- | -- | -- | -- | -- | -- | 356 | 5.430 | 75.936 | 0.082 | 0.040 | 0.89 | 356 | 5.430 | 75.936 | 0.590 | 0.087 | 0.078 | 0.034 | 0.11 |
| 80420_wint_10_11 | TP | 200 | 9.605 | 87.780 | 2.436 | 0.054 | -- | -- | -- | -- | -- | -- | -- | -- | -- | -- | -- | -- | -- | -- | -- |
| 80420_wint_11_12 | TEXP | -- | -- | -- | -- | -- | -- | 258 | 7.295 | 103.454 | 0.086 | 0.058 | 0.74 | 258 | 7.295 | 103.454 | 0.612 | 0.090 | 0.081 | 0.059 | 0.26 |
| 89730_wint_09_10 | TEXP | -- | -- | -- | -- | -- | -- | 385 | 4.447 | 51.693 | 0.090 | 0.028 | 1.00 | 523 | 1.446 | 51.693 | 1.000 | 0.096 | 0.087 | 0.023 | 0.00 |
| 89730_wint_10_11 | TEXP | -- | -- | -- | -- | -- | -- | 456 | 1.595 | 104.240 | 0.088 | 0.026 | 0.73 | 457 | 1.584 | 104.240 | 0.632 | 0.092 | 0.082 | 0.026 | 0.27 |
| 89730_wint_11_12 | TEXP | 186 | 11.600 | 52.389 | 2.605 | 0.056 | -- | 251 | 8.420 | 52.389 | 0.107 | 0.038 | 0.95 | 220 | 9.695 | 52.389 | 1.000 | 0.113 | 0.102 | 0.047 | 0.05 |
| 89731_wint_09_10 | TEXP | 204 | 15.541 | 60.939 | 2.763 | 0.062 | -- | 225 | 14.228 | 60.939 | 0.094 | 0.041 | 0.97 | 225 | 14.228 | 60.939 | 1.000 | 0.099 | 0.089 | 0.050 | 0.03 |
| 89731_wint_10_11 | TEXP | 187 | 10.327 | 46.942 | 2.099 | 0.045 | -- | 457 | 1.299 | 46.942 | 0.098 | 0.030 | 1.00 | 282 | 6.527 | 46.942 | 1.000 | 0.109 | 0.098 | 0.033 | 0.01 |
| 89731_wint_11_12 | TEXP | -- | -- | -- | -- | -- | -- | 469 | 1.630 | 64.38 | 0.089 | 0.038 | 0.94 | 488 | 1.296 | 64.377 | 1.000 | 0.092 | 0.083 | 0.041 | 0.07 |
