# Supplementary material for: Scavengers on the Move: Behavioural Changes in Foraging Search Patterns during the Annual Cycle
Source: PLoS One. 2013 Jan 23;8(1):e54352. doi: 10.1371/journal.pone.0054352 (PMC3553087; doi:10.1371/journal.pone.0054352)
Supplement: Appendix S1 — Probability density function (pdf), cumulative density function (cdf) and complementary density function (CDF) for the Truncated Exponential distribution. (PDF) [file pone.0054352.s008.pdf]

APPENDIX S1

Probability density function (pdf), cumulative density function (cdf) and complementary density function (CDF) for the Truncated Exponential distribution.

Consider the exponential distribution, with the normalization constant C:

$$pdf(x) = C.\ e^{-\lambda.x} \qquad \text{(eq. 1)}$$

Then we need to calculate C. We start with the normalization requirement that the integral of the probability density function of the exponential distribution must sum one between the lower (a = x_{min}) and upper (b = x_{max}) truncation bounds:

$$1 = \int_a^b pdf(x)\ dx = \int_a^b C.e^{-\lambda.x} \qquad \text{(eq. 2)}$$

Solving equation 2:

$$1 = C.\left[-\frac{1}{\lambda}.e^{-\lambda.x} + k\right]_a^b \qquad \text{(eq. 3)}$$

where k is a constant,

$$1 = C.\left[\left(-\frac{1}{\lambda}.e^{-\lambda.b} + k\right) - \left(-\frac{1}{\lambda}.e^{-\lambda.a} + k\right)\right] \qquad \text{(eq. 4)}$$

$$1 = C.\frac{1}{\lambda}.\left[e^{-\lambda.a} - e^{-\lambda.b}\right] \qquad \text{(eq. 5)}$$

We finally obtain C as follows:

$$C = \frac{\lambda}{e^{-\lambda.a} - e^{-\lambda.b}} \qquad \text{(eq. 6)}$$

Then, if we replace C in the eq. 1 by the expression of the normalization constant obtained in the eq. 6, we obtain the probability density function for the truncated exponential, where a is the lower bound (x_{min}) and b is the upper bound (x_{max}):

$$pdf\ (x) = \frac{\lambda}{e^{-\lambda.a} - e^{-\lambda.b}} \cdot \ e^{-\lambda.x} \qquad \text{(eq. 7)}$$

To obtain the cumulative density function we replace the normalization constant C by its developed expression obtained in eq.6:

$$cdf\ (x) = \frac{\lambda}{e^{-\lambda.a} - e^{-\lambda.b}} \cdot \frac{1}{-\lambda} \cdot e^{-\lambda.x} + \ k \qquad \text{(eq. 8)}$$

We can calculate k solving eq.8 in two ways: i) $cdf(x) = 0$ when $x = a$; or ii) $cdf(x) = 1$ when $x = b$, then solving the first way:

$$cdf(a) = \frac{\lambda}{e^{-\lambda.a} - e^{-\lambda.b}} \cdot \frac{1}{-\lambda} \cdot e^{-\lambda.a} + \ k = 0 \qquad \text{(eq. 9)}$$

$$\frac{\lambda}{-\lambda} \cdot \frac{1}{e^{-\lambda.a} - e^{-\lambda.b}} \cdot e^{-\lambda.a} = -k \qquad \text{(eq. 10)}$$

$$k = \frac{e^{-\lambda.a}}{e^{-\lambda.a} - e^{-\lambda.b}} \qquad \text{(eq. 11)}$$

Replacing k in eq.8 by the expression obtained in the eq. 11 we get:

$$cdf\ (x) = \frac{\lambda}{e^{-\lambda.a} - e^{-\lambda.b}} \cdot \frac{1}{-\lambda} \cdot e^{-\lambda.x} + \frac{e^{-\lambda.a}}{e^{-\lambda.a} - e^{-\lambda.b}} \qquad \text{(eq. 12)}$$

and simplifying:

$$cdf\ (x) = \frac{e^{-\lambda.a}}{e^{-\lambda.a} - e^{-\lambda.b}} - \frac{e^{-\lambda.x}}{e^{-\lambda.a} - e^{-\lambda.b}} \qquad \text{(eq. 13)}$$

The complementary cumulative density function (CDF) is:

$$CDF\ (x) = 1 - cdf\ (x) = 1 - \left[\frac{e^{-\lambda.a}}{e^{-\lambda.a} - e^{-\lambda.b}} - \frac{e^{-\lambda.x}}{e^{-\lambda.a} - e^{-\lambda.b}}\right] \qquad \text{(eq. 14)}$$
