# Supplementary material for: Scavengers on the Move: Behavioural Changes in Foraging Search Patterns during the Annual Cycle
Source: PLoS One. 2013 Jan 23;8(1):e54352. doi: 10.1371/journal.pone.0054352 (PMC3553087; doi:10.1371/journal.pone.0054352)
Supplement: Appendix S2 — Process diagram of the methods used in this study. (PDF) [file pone.0054352.s009.pdf]

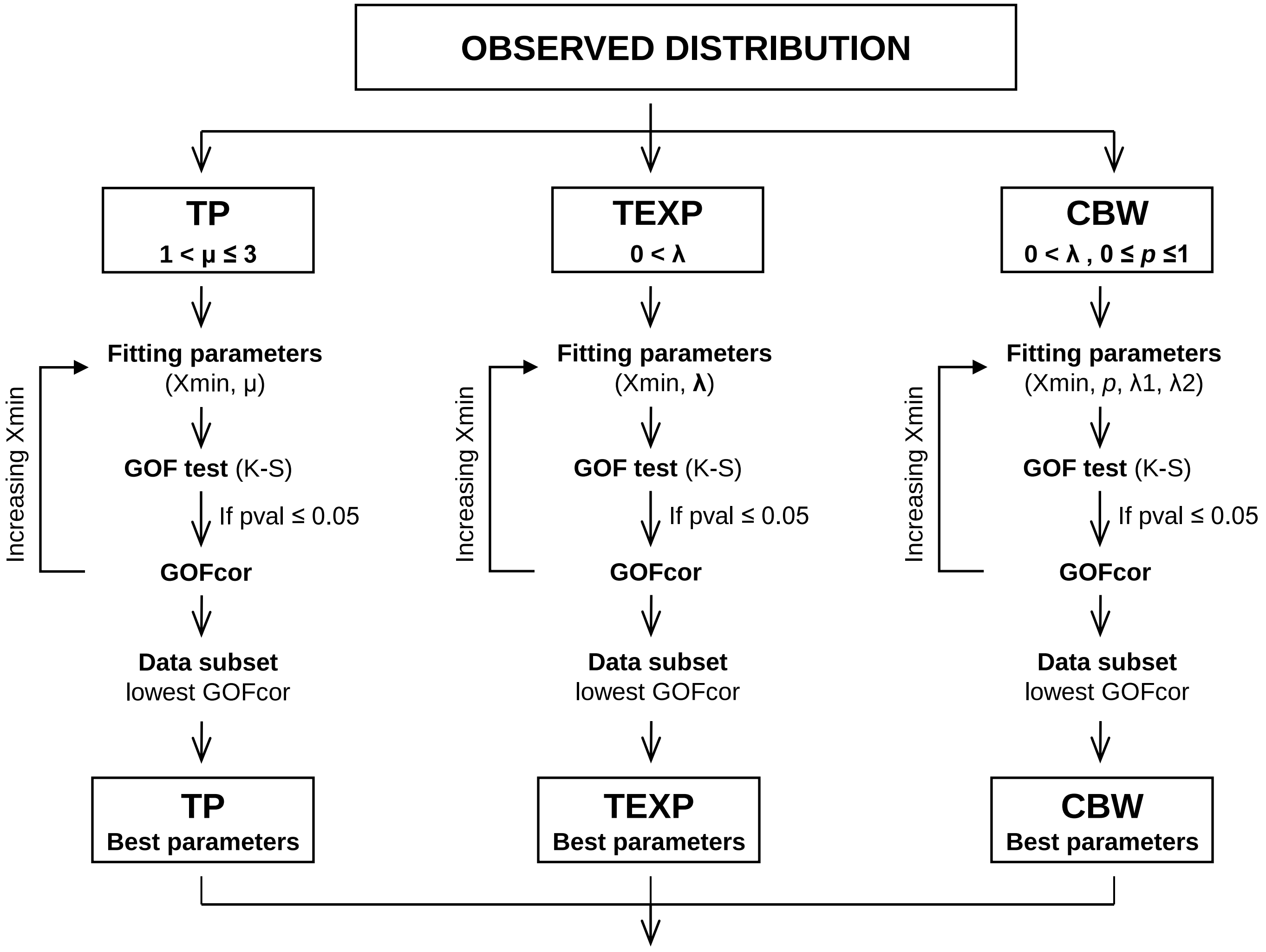

MODEL SELECTION CRITERIA

If **any** model is significant → **No** model is selected

Only **one** significant model → This **one** is the selected model

Two significant models → **wAIC and Truth Table**

Three significant models → **wAIC and Truth Table**
Between two models with lowest GOFcor

The "truth-table"

**wAIC values**				
Fitted model 1	Competing model 2	Fitted model 2	Competing model 1	WINNER
1	0	0	1	model 1
0	1	1	0	model 2
1	0	1	0	lowest GOFcor
0	1	0	1	mixed model
